# (*Z*)-3-Hy­droxy-4-(4-meth­oxy­phen­yl)but-3-en-2-one

**DOI:** 10.1107/S1600536813002262

**Published:** 2013-02-09

**Authors:** Wei Fang, Jun-Ping Hu, Mei Wang, Mu-Zi Chen

**Affiliations:** a Analysis and Testing Center, Dushu Lake Campus, Suzhou University, Suzhou 215123, People’s Republic of China; b Department of Chemistry, Handan College, Hebei Handan 056002, People’s Republic of China

## Abstract

The title compound, C_11_H_12_O_3_, is potentially a butane-2,3-dione derivative but exists in the enol form in the solid state. In the mol­ecule, the 3-hy­droxy­but-3-en-2-one, benzene and methoxyl fragments are almost co-planar. The 3-hy­droxy­but-3-en-2-one fragment is almost planar with an r.m.s. deviation of 0.040 Å. The dihedral angle between this plane and that of the benzene ring is 5.88 (4)°. The 4-meth­oxy group also lies close to the benzene ring plane, with deviations of 0.0206 (11) Å for the O and 0.087 (2) Å for methyl C atoms. Hence, the whole mol­ecule is almost planar with an r.m.s. deviation of 0.0617 Å from a plane through all 14 non-H atoms. In the crystal, the molecules are linked by O—H⋯O hydrogen bonds, generating [010] chains.

## Related literature

The synthesis of the compound is described by Wang & Huang (2010 ▶). For applications of aromatic ketones as fragrances, see: Tong *et al.* (2009 ▶). For the relationship between structure and fragrance, see: Griesbeck *et al.* (2012 ▶). For related structures and details of their synthesis, see: Yamane *et al.* (2005 ▶); Si *et al.* (1990 ▶); Salimbeni *et al.* (1987 ▶); Mosrin *et al.* (2009 ▶). For standard bond lengths, see: Allen *et al.* (1987 ▶).
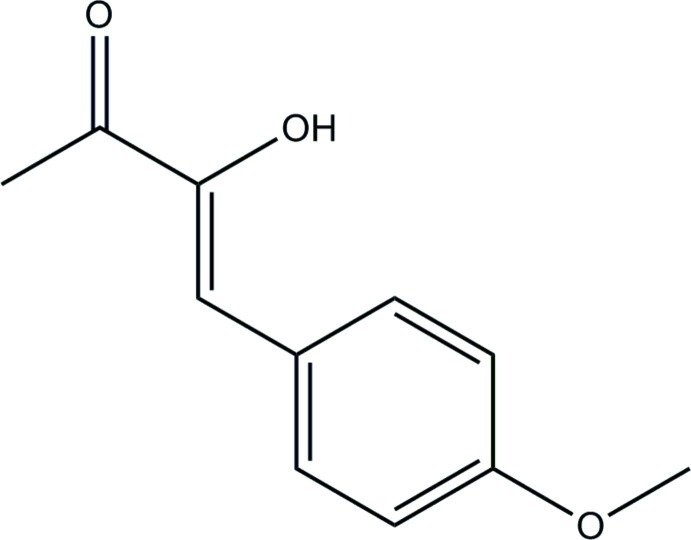


## Experimental

### 

#### Crystal data

C_11_H_12_O_3_
*M* *_r_* = 192.21
Monoclinic, 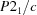

*a* = 18.8076 (13) Å
*b* = 5.3007 (4) Å
*c* = 10.1439 (8) Å
β = 103.425 (7)°
*V* = 983.65 (13) Å^3^
*Z* = 4
Mo *K*α radiation
μ = 0.09 mm^−1^
*T* = 223 K
0.50 × 0.40 × 0.35 mm

#### Data collection

Agilent Xcalibur (Atlas CCD, Gemini) diffractometer
Absorption correction: multi-scan (*CrysAlis PRO*; Agilent, 2012 ▶) *T*_min_ = 0.915, *T*_max_ = 1.000
6213 measured reflections
1829 independent reflections
1505 reflections with *I* > 2σ(*I*)
*R*_int_ = 0.024

#### Refinement

*R*[*F* ^2^ > 2σ(*F* ^2^)] = 0.042
*wR*(*F* ^2^) = 0.113
*S* = 1.01
1829 reflections
130 parameters
H-atom parameters constrained
Δρ_max_ = 0.17 e Å^−3^
Δρ_min_ = −0.13 e Å^−3^

### 

Data collection: *CrysAlis PRO* (Agilent, 2012 ▶); cell refinement: *CrysAlis PRO*; data reduction: *CrysAlis PRO*; program(s) used to solve structure: *SHELXS97* (Sheldrick, 2008 ▶); program(s) used to refine structure: *SHELXL97* (Sheldrick, 2008 ▶); molecular graphics: *DIAMOND* (Brandenburg, 2004 ▶); software used to prepare material for publication: *publCIF* (Westrip, 2010 ▶).

## Supplementary Material

Crystal structure: contains datablock(s) I. DOI: 10.1107/S1600536813002262/sj5292sup1.cif

Structure factors: contains datablock(s) I. DOI: 10.1107/S1600536813002262/sj5292Isup2.hkl

Supplementary material file. DOI: 10.1107/S1600536813002262/sj5292Isup3.cdx

Supplementary material file. DOI: 10.1107/S1600536813002262/sj5292Isup4.cml

Additional supplementary materials:  crystallographic information; 3D view; checkCIF report

## Figures and Tables

**Table 1:** Hydrogen-bond geometry (Å, °)

*D*—H⋯*A*	*D*—H	H⋯*A*	*D*⋯*A*	*D*—H⋯*A*
O2—H2⋯O1^i^	0.82	2.27	3.0315 (17)	154

Symmetry code: (i) 
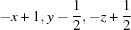
.

**Fig. 1.**
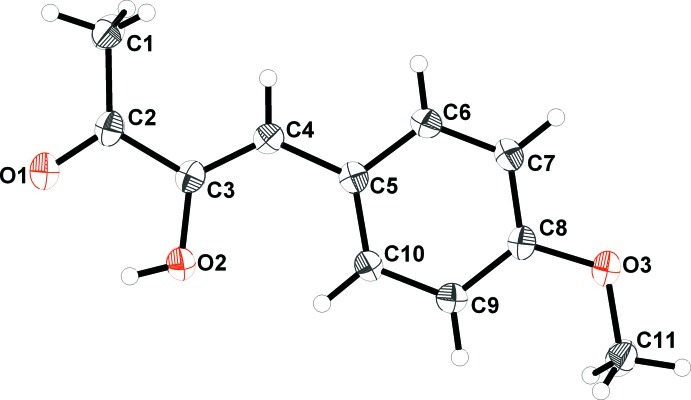
The molecular structure of the title compound, with ellipsoids drawn at the 30% probability level. Hydrogen atoms are presented as a small spheres of arbitrary radius.

**Fig. 2.**
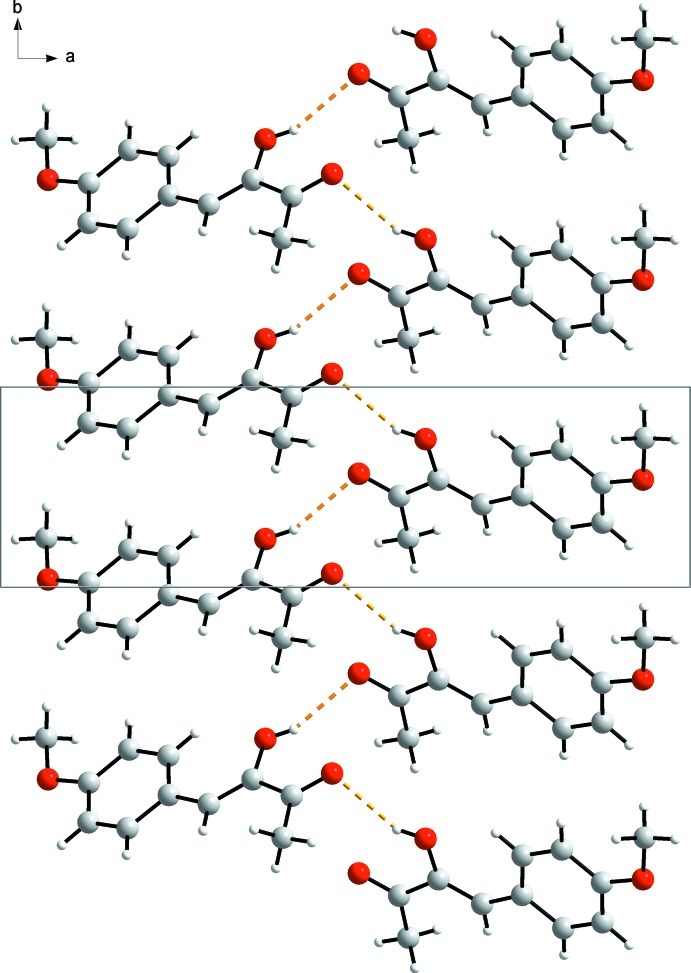
The title compound forms a helical chain along the b axis through intermolecular O—H···O hydrogen bonds.

## supplementary crystallographic information

### Comment

Aromatic ketone compounds have attracted much attention due to their applications in fragrances and perfume technology (Tong *et al.,* 2009). Studying the relationship between molecular structures and their fragrant properties remains a challenge (Griesbeck *et al.,* 2012). Understanding the molecular structure in detail will help to design more compounds with potential as fragrances. As a part of our work in this area (Wang & Huang, 2010), a new aromatic diketone compound, (*Z*)-3-Hydroxy-4- (4-methoxyphenyl)but-3-en-2-one (Figure 1), was synthesized and its molecular structure is reported here. The title compound crystallizes with one unique molecule in the asymmetric unit. In the molecule, the 3-hydroxybut-3-en-2-one, phenyl and methoxyl fragments are close to co-planar. The O1, O2 and C1—C4 atoms of the 3-hydroxybut-3-en-2-one fragment form a plane with an rms deviation of 0.0359 Å. The dihedral angle between this plane and the benzene ring plane is 5.88 (4)°. The 4-methoxyl group lies close to the benzene ring plane, with deviations of 0.0206 (11) Å for O3 and 0.087 (2) Å for C11. The dihedral angle between benzene ring and plane of 4-methoxyl group (O3—C11—C8) is 5.88 (4)°. Hence the whole molecule is close to planar, with an rms deviation of 0.0496 Å from the plane through all non-hydrogen atoms in the molecule. A characteristic of title compound is that it adopts the enol form with a C3=C4 distance 1.341 (2) Å. The conformation about the C3=C4 bond is *Z*. Bond lengths (Allen *et al.*, 1987) and angles are within normal ranges and are comparable to those found in related structures (Yamane, *et al.*, 2005; Si *et al.*, 1990; Salimbeni *et al.*, 1987; Mosrin *et al.*, 2009). Intermolecular O—H···O hydrogen bonds arrange the molecules into a helical chain along the *b* axis (Figure 2).

### Experimental

A mixture of hydroxy-acetone and anisaldehyde was added dropwise into warm hydrochloric acid at 50° C. The resulting solution was heated to 82° C for two hours, then cooled to room temperature as described by Wang & Huang (2010). A white amorphous product was obtained after filtration. Yellow crystals of title compound, suitable for X-ray analysis, were recrystallized from absolute ethanol over two weeks.

### Refinement

All H atoms were located in calculated positions with the aromatic C–H = 0.93 Å, methyl C–H = 0.96 Å hydroxy O–H = 0.82 Å and displacement parameters set at 1.2U_eq_ (aromatic) and 1.5U_eq_ (methyl and OH) of the parent atom.

### Crystal data

**Table:**

C_11_H_12_O_3_	*F*(000) = 408
*M**_r_* = 192.21	*D*_x_ = 1.298 Mg m^−^^3^
Monoclinic, *P*2_1_/*c*	Mo *K*α radiation, λ = 0.71073 Å
Hall symbol: -P 2ybc	Cell parameters from 1975 reflections
*a* = 18.8076 (13) Å	θ = 3.3–29.4°
*b* = 5.3007 (4) Å	µ = 0.09 mm^−^^1^
*c* = 10.1439 (8) Å	*T* = 223 K
β = 103.425 (7)°	Block, yellow
*V* = 983.65 (13) Å^3^	0.50 × 0.40 × 0.35 mm
*Z* = 4	

### Data collection

**Table:**

Agilent Xcalibur (Atlas CCD, Gemini) diffractometer	1829 independent reflections
Radiation source: fine-focus sealed tube	1505 reflections with *I* > 2σ(*I*)
Graphite monochromator	*R*_int_ = 0.024
ω scans	θ_max_ = 25.5°, θ_min_ = 3.3°
Absorption correction: multi-scan (*CrysAlis PRO*; Agilent, 2012)	*h* = −22→22
*T*_min_ = 0.915, *T*_max_ = 1.000	*k* = −6→6
6213 measured reflections	*l* = −12→12

### Refinement

**Table:**

Refinement on *F*^2^	Primary atom site location: structure-invariant direct methods
Least-squares matrix: full	Secondary atom site location: difference Fourier map
*R*[*F*^2^ > 2σ(*F*^2^)] = 0.042	Hydrogen site location: inferred from neighbouring sites
*wR*(*F*^2^) = 0.113	H-atom parameters constrained
*S* = 1.01	*w* = 1/[σ^2^(*F*_o_^2^) + (0.0537*P*)^2^ + 0.2906*P*] where *P* = (*F*_o_^2^ + 2*F*_c_^2^)/3
1829 reflections	(Δ/σ)_max_ < 0.001
130 parameters	Δρ_max_ = 0.17 e Å^−^^3^
0 restraints	Δρ_min_ = −0.13 e Å^−^^3^

### Special details

**Table:**

Geometry. All e.s.d.'s (except the e.s.d. in the dihedral angle between two l.s. planes) are estimated using the full covariance matrix. The cell e.s.d.'s are taken into account individually in the estimation of e.s.d.'s in distances, angles and torsion angles; correlations between e.s.d.'s in cell parameters are only used when they are defined by crystal symmetry. An approximate (isotropic) treatment of cell e.s.d.'s is used for estimating e.s.d.'s involving l.s. planes.
Refinement. Refinement of *F*^2^ against ALL reflections. The weighted *R*-factor *wR* and goodness of fit *S* are based on *F*^2^, conventional *R*-factors *R* are based on *F*, with *F* set to zero for negative *F*^2^. The threshold expression of *F*^2^ > σ(*F*^2^) is used only for calculating *R*-factors(gt) *etc*. and is not relevant to the choice of reflections for refinement. *R*-factors based on *F*^2^ are statistically about twice as large as those based on *F*, and *R*- factors based on ALL data will be even larger.

### Fractional atomic coordinates and isotropic or equivalent isotropic displacement parameters (Å^2^)

**Table:**

	*x*	*y*	*z*	*U*_iso_*/*U*_eq_	
O1	0.48064 (6)	0.4343 (3)	0.31418 (12)	0.0567 (4)	
O2	0.38385 (6)	0.2608 (2)	0.10575 (12)	0.0548 (4)	
H2	0.4252	0.2145	0.1431	0.082*	
O3	0.06871 (6)	0.4633 (2)	−0.32947 (11)	0.0527 (4)	
C1	0.40895 (10)	0.7551 (4)	0.38093 (17)	0.0556 (5)	
H1A	0.3996	0.9078	0.3289	0.083*	
H1B	0.4513	0.7774	0.4538	0.083*	
H1C	0.3675	0.7155	0.4173	0.083*	
C2	0.42216 (9)	0.5450 (3)	0.29207 (16)	0.0421 (4)	
C3	0.36530 (8)	0.4639 (3)	0.17241 (15)	0.0396 (4)	
C4	0.30191 (8)	0.5866 (3)	0.12778 (15)	0.0396 (4)	
H4	0.2938	0.7202	0.1818	0.048*	
C5	0.24383 (8)	0.5416 (3)	0.00700 (15)	0.0372 (4)	
C6	0.18417 (9)	0.7074 (3)	−0.02124 (16)	0.0425 (4)	
H6	0.1830	0.8411	0.0375	0.051*	
C7	0.12736 (9)	0.6784 (3)	−0.13315 (16)	0.0446 (4)	
H7	0.0886	0.7918	−0.1494	0.053*	
C8	0.12795 (8)	0.4798 (3)	−0.22176 (15)	0.0394 (4)	
C9	0.18628 (9)	0.3141 (3)	−0.19755 (16)	0.0443 (4)	
H9	0.1872	0.1815	−0.2572	0.053*	
C10	0.24337 (9)	0.3452 (3)	−0.08458 (16)	0.0439 (4)	
H10	0.2823	0.2323	−0.0695	0.053*	
C11	0.06626 (10)	0.2552 (4)	−0.41902 (18)	0.0573 (5)	
H11A	0.0221	0.2630	−0.4891	0.086*	
H11B	0.0672	0.1005	−0.3694	0.086*	
H11C	0.1077	0.2616	−0.4590	0.086*	

### Atomic displacement parameters (Å^2^)

**Table:**

	*U*^11^	*U*^22^	*U*^33^	*U*^12^	*U*^13^	*U*^23^
O1	0.0445 (7)	0.0652 (8)	0.0530 (7)	0.0081 (6)	−0.0037 (5)	−0.0056 (6)
O2	0.0446 (7)	0.0531 (8)	0.0577 (7)	0.0111 (6)	−0.0064 (5)	−0.0111 (6)
O3	0.0452 (7)	0.0585 (8)	0.0466 (7)	0.0075 (6)	−0.0052 (5)	−0.0051 (6)
C1	0.0533 (10)	0.0689 (13)	0.0415 (9)	0.0015 (9)	0.0045 (8)	−0.0094 (9)
C2	0.0400 (9)	0.0485 (10)	0.0366 (8)	−0.0028 (8)	0.0067 (6)	0.0051 (7)
C3	0.0402 (8)	0.0408 (9)	0.0372 (8)	−0.0031 (7)	0.0076 (7)	0.0013 (7)
C4	0.0398 (8)	0.0415 (9)	0.0373 (8)	−0.0018 (7)	0.0084 (6)	−0.0014 (7)
C5	0.0352 (8)	0.0368 (8)	0.0395 (8)	−0.0010 (7)	0.0085 (6)	0.0029 (6)
C6	0.0460 (9)	0.0385 (9)	0.0425 (9)	0.0051 (7)	0.0092 (7)	−0.0028 (7)
C7	0.0420 (9)	0.0435 (9)	0.0461 (9)	0.0110 (7)	0.0060 (7)	0.0033 (7)
C8	0.0365 (8)	0.0436 (9)	0.0361 (8)	−0.0006 (7)	0.0044 (6)	0.0044 (7)
C9	0.0427 (9)	0.0432 (9)	0.0447 (9)	0.0035 (7)	0.0056 (7)	−0.0063 (7)
C10	0.0372 (8)	0.0428 (9)	0.0485 (9)	0.0075 (7)	0.0032 (7)	−0.0037 (7)
C11	0.0547 (11)	0.0566 (12)	0.0512 (10)	−0.0028 (9)	−0.0067 (8)	−0.0082 (9)

### Geometric parameters (Å, º)

**Table:**

O1—C2	1.2206 (19)	C5—C10	1.394 (2)
O2—C3	1.3592 (19)	C5—C6	1.401 (2)
O2—H2	0.8200	C6—C7	1.375 (2)
O3—C8	1.3703 (18)	C6—H6	0.9300
O3—C11	1.423 (2)	C7—C8	1.386 (2)
C1—C2	1.490 (2)	C7—H7	0.9300
C1—H1A	0.9600	C8—C9	1.382 (2)
C1—H1B	0.9600	C9—C10	1.386 (2)
C1—H1C	0.9600	C9—H9	0.9300
C2—C3	1.483 (2)	C10—H10	0.9300
C3—C4	1.341 (2)	C11—H11A	0.9600
C4—C5	1.459 (2)	C11—H11B	0.9600
C4—H4	0.9300	C11—H11C	0.9600
			
C3—O2—H2	109.5	C7—C6—H6	119.0
C8—O3—C11	117.29 (13)	C5—C6—H6	119.0
C2—C1—H1A	109.5	C6—C7—C8	119.92 (15)
C2—C1—H1B	109.5	C6—C7—H7	120.0
H1A—C1—H1B	109.5	C8—C7—H7	120.0
C2—C1—H1C	109.5	O3—C8—C9	124.47 (15)
H1A—C1—H1C	109.5	O3—C8—C7	116.00 (14)
H1B—C1—H1C	109.5	C9—C8—C7	119.54 (14)
O1—C2—C3	117.32 (15)	C8—C9—C10	120.12 (15)
O1—C2—C1	121.21 (15)	C8—C9—H9	119.9
C3—C2—C1	121.47 (15)	C10—C9—H9	119.9
C4—C3—O2	121.79 (14)	C9—C10—C5	121.55 (15)
C4—C3—C2	123.48 (15)	C9—C10—H10	119.2
O2—C3—C2	114.64 (14)	C5—C10—H10	119.2
C3—C4—C5	129.63 (15)	O3—C11—H11A	109.5
C3—C4—H4	115.2	O3—C11—H11B	109.5
C5—C4—H4	115.2	H11A—C11—H11B	109.5
C10—C5—C6	116.83 (14)	O3—C11—H11C	109.5
C10—C5—C4	124.68 (14)	H11A—C11—H11C	109.5
C6—C5—C4	118.49 (14)	H11B—C11—H11C	109.5
C7—C6—C5	122.04 (15)		

### Hydrogen-bond geometry (Å, º)

**Table:**

*D*—H···*A*	*D*—H	H···*A*	*D*···*A*	*D*—H···*A*
O2—H2···O1^i^	0.82	2.27	3.0315 (17)	154

Symmetry code: (i) −*x*+1, *y*−1/2, −*z*+1/2.
